# Cross‐elicitation responses to 2‐methoxymethyl‐*p*‐phenylenediamine in *p*‐phenylenediamine‐allergic individuals: Results from open use testing and diagnostic patch testing

**DOI:** 10.1111/cod.13078

**Published:** 2018-08-07

**Authors:** Marie L. Schuttelaar, Daan Dittmar, Johannes G. M. Burgerhof, Brunhilde Blömeke, Carsten Goebel

**Affiliations:** ^1^ Department of Dermatology University of Groningen, University Medical Center Groningen Groningen The Netherlands; ^2^ Department of Epidemiology University of Groningen, University Medical Center Groningen Groningen The Netherlands; ^3^ Department of Environmental Toxicology Trier University Trier Germany; ^4^ Department of Toxicology Coty Darmstadt Germany

**Keywords:** contact allergy, allergic contact dermatitis, diagnostic patch tests, open use test, hair dyes, *p*‐phenylenediamine, 2‐methoxymethyl‐*p*‐phenylenediamine

## Abstract

**Background:**

Allergic contact dermatitis caused by *p*‐phenylenediamine (PPD) is a health concern for hair dye users. Because of its lower sensitization potency, the PPD derivative 2‐methoxymethyl‐*p*‐phenylenediamine (ME‐PPD) has been developed as an alternative hair dye for primary prevention. However, cross‐elicitation responses can occur in PPD‐allergic subjects.

**Objectives:**

To compare cross‐elicitation responses to ME‐PPD in open use and diagnostic patch testing of PPD‐allergic subjects with hair dye‐related allergic contact dermatitis.

**Methods:**

Reactions to ME‐PPD were investigated in 25 PPD‐allergic subjects by performing (1) 45‐minute open use testing with a hair dye containing 2.0% of either ME‐PPD or PPD, and (2) patch testing with increasing ME‐PPD concentrations (0.1%–2.0% pet.).

**Results:**

Of the 25 PPD‐allergic subjects, 21 (84%) reacted to open use testing with a hair dye containing 2.0% PPD, and testing with 2.0% ME‐PPD led to cross‐elicitation in 12 (48%). When patch tested with increasing ME‐PPD concentrations, 13 (52%) cross‐reacted at 0.1% (lowest dose) and 21 (84%) at 2.0% (highest dose), indicating decreased reactivity as compared with published PPD dose‐response data.

**Conclusion:**

In line with the decreased cross‐reactivity of ME‐PPD in hair dye open use testing, PPD‐allergic subjects show an attenuated cross‐elicitation dose response to ME‐PPD in patch testing.

## 1 INTRODUCTION

*p*‐Phenylenediamine (PPD, 1,4‐diaminobenzene, CAS no. 106‐50‐3) is a hair dye molecule with good hair‐colouring performance, but is also an important contact allergen associated with hair dye‐related allergic contact dermatitis. Historically, attempts have been made to develop hair dye molecules that keep the balance between good hair‐dyeing performance and sufficiently low skin sensitization potency to avoid induction under use conditions. The resulting PPD derivatives were toluene‐2,5‐diamine (TDA, 1,4‐diamino‐2‐methylbenzene, CAS no. 95‐70‐5; synonym *p*‐toluenediamine) and hydroxyethyl‐*p*‐phenylenediamine sulfate (HE‐PPD, CAS no. 93841‐25‐9): TDA has good performance, but may lead to the induction of sensitization under use conditions, owing to its strong to extreme sensitization potency, as determined with the murine local lymph node assay (LLNA) and the guinea‐pig maximization test. HE‐PPD has limited performance and strong sensitization potency (as determined with the LLNA).^1^, ^2^, ^3^

More recently, 2‐methoxymethyl‐*p*‐phenylenediamine (ME‐PPD, CAS no. 337906‐36‐2) has been developed by introducing a methoxymethyl side‐chain into PPD, resulting in a hair‐dyeing performance equivalent to that of PPD and TDA. Analysis of the skin sensitization potency of ME‐PPD in in vitro studies indicated a lower skin sensitization potency than that of PPD and TDA. In vivo, the LLNA showed a moderate skin sensitization potency of ME‐PPD. Therefore, induction of skin sensitization has been considered to be unlikely when ME‐PPD is used to replace PPD or TDA in hair dyes.^3^

Accordingly, ME‐PPD has been developed for the prevention of skin sensitization, and not for individuals who have already been sensitized to other hair dye precursors, such as PPD and TDA. However, it is known that many individuals who are allergic to PPD or TDA continue to dye their hair, and they may use hair dye products that contain ME‐PPD instead of PPD or TDA.^4^ Therefore, the current study investigated cross‐elicitation responses to an ME‐PPD‐containing hair dye under open use test conditions in PPD‐allergic individuals with a history of hair dye‐related allergic contact dermatitis. Furthermore, their cross‐elicitation dose response to ME‐PPD was determined under diagnostic patch test conditions, and compared with PPD elicitation dose‐response data previously published by Søsted et al.^5^

## 2 METHODS

Twenty‐five adult individuals with a previously documented positive patch test reaction to PPD 90 μg/cm^2^ (TRUE Test; SmartPractice Europe, Reinbek, Germany) or PPD 1% pet. (Chemotechnique Diagnostics, Vellinge, Sweden) and a history of allergic contact dermatitis caused by hair dye were included. The exclusion criteria were as follows: skin anomalies or active dermatitis on the volar aspects of the forearms or on the back, and the use of immunosuppressive medication (including, but not limited to, oral corticosteroids, cyclosporine, azathioprine, and methotrexate) during the 4 weeks prior to inclusion. All tests were performed at the Department of Dermatology, University Medical Center Groningen, The Netherlands, and assessed according to ESCD guidelines on day (D) 2, D3, and D7.^6^ Of the 25 subjects included in this present study, 8 had participated in a previous ME‐PPD open use test study.^7^ In the previous study, 6 of these 8 reacted positively to ME‐PPD under hair dye conditions (open use test), and the other 2 did not. The study was approved by the local ethics committee of the University Medical Center Groningen.

### 2.1 Open use testing

Open use tests were performed on the volar aspects of both forearms. The patch test preparations, the vehicle (Koleston Perfect formula without fragrance) containing the hair dye precursors (PPD 4.0% or ME‐PPD 4.0%, free base) and couplers (1.9% 2‐methyl‐5‐hydroxyethylaminophenol; 3.6% 2‐methylresorcinol) and the hydrogen peroxide‐based developer solution (6.0% wt/wt Welloxon) were provided by Procter & Gamble Service (now represented by Coty, Darmstadt, Germany). The couplers were selected on the basis of their negligible sensitization potency as determined with the LLNA, each with an EC3 greater than 50.^8^, ^9^ The hair dye test product was always freshly prepared by mixing the tint (containing PPD or ME‐PPD, and the couplers) with the hydrogen peroxide solution by use of a small wooden stick (1:1, 90 μL each), resulting in solutions containing 2.0% PPD and 2.0% ME‐PPD, respectively. A dye‐free test product, also mixed with hydrogen peroxide as described above, was used as a negative control. A 100‐μL aliquot of the final PPD‐containing or ME‐PPD‐containing product was applied directly to the volar forearm with a micropipette in a 3.8‐cm^2^ area marked by a round adhesive tape with a diameter of 22 mm. PPD was tested on the volar aspect of 1 arm, and ME‐PPD and the negative control were tested on the other, so that an extreme positive reaction to PPD would have no influence on a possible elicitation response to ME‐PPD. The contours of the round adhesive tape were marked with a Chemo Skin Marker (Chemotechnique Diagnostics), in order to enable recognition of the test site at follow‐up. The test areas were rinsed off with water after 45 minutes of application to simulate hair‐dyeing conditions.

### 2.2 Diagnostic patch testing

Patch testing was performed on the back with ME‐PPD 0.1%, 0.25%, 0.5%, 1.0% and 2.0% pet. in Van der Bend chambers (Van der Bend, Brielle, The Netherlands), fixed with Fixomull Stretch (BSN Medical, Hamburg, Germany). In the first 10 subjects, ME‐PPD was only patch tested in concentrations of 0.5%, 1.0% and 2.0% pet., after which the protocol was adapted to also include patch testing with ME‐PPD 0.1% and 0.25% pet. The first 10 subjects were recalled for testing with these 2 concentrations, and all except for 2 subjects were additionally tested.

### 2.3 Evaluation and statistics

Responses to the open use test were recorded at 60 minutes after application (15 minutes after rinsing of the area), and, together with the patch test readings, on D2, D3, and D7, and graded according to ESCD guidelines.^6^ For data analysis, the maximum open use test/patch test reactions were aggregated as the outcome.

Results are presented as the proportion of PPD‐allergic patients who responded with a cross‐elicitation response to ME‐PPD as indicated by a positive patch test reaction for each dose. Binary logistic regression was performed, and a dose‐response curve was plotted in order to investigate the threshold dose for cross‐elicitation for different proportions of the study population. The probability of a positive response *P*(*x*), where *x* represents the natural logarithm of the dose, for a given dose is as follows:

$$P\left(x\right)=\frac{\exp\left(b0+b1^{*}x\right)}{1+\exp\left(b0+b1^{*}x\right)}$$

The effective dose (ED*y*), that is, the hypothetical dose at which a proportion *y* of the 25 PPD‐positive patients have a positive cross‐elicitation response to ME‐PPD, was calculated for 50%, 75% and 90% of the patients. For example, ED50 is the expected dose at which 50% of the 25 PPD‐positive patients will have a positive response to ME‐PPD. The confidence interval (CI) for each ED*y* is given. The dose‐response curve is presented for all 25 PPD‐positive patients, and compared with the dose‐response curve for PPD as reported by Søsted et al.^5^ Dose‐response curves were generated by the use of graphpad prism 7.03, with non‐linear regression with curve fitting (GraphPad Software, La Jolla, California).

## 3 RESULTS

### 3.1 Cross‐elicitation response to ME‐PPD‐containing hair dye in open use testing

The strengths of the original patch test reactions to PPD were weak (+) positive (*n* = 5), strong (++) positive (*n* = 12), and extreme (+++) positive (*n* = 8). The characteristics of the 25 subjects are shown in Table 1. A detailed overview of the individual open use and diagnostic patch test results obtained from the 25 PPD‐allergic subjects is shown in Table 2. Twelve of the 25 subjects (48%, 95%CI: 28%‐68%) showed cross‐elicitation reactions to the open use test with hair dye containing 2.0% ME‐PPD (Table 2), whereas 13 of 25 (52%) showed no cross‐elicitation response. In the 12 of 25 subjects reacting to ME‐PPD, the strengths of the reactions to the ME‐PPD open use test were generally weaker than those to the 2.0% PPD open use test, with 6 of 12 having weak positive reactions, 5 of 12 having strong positive reactions and 1 of 12 having an extreme positive reaction to ME‐PPD, as compared with 2 of 21 having weak positive reactions, 10 of 21 having strong positive reactions and 9 of 21 having extreme positive reactions to PPD. Correspondingly, 11 of 25 had reduced strengths of reaction to the ME‐PPD open use test as compared with the PPD open use test, not counting 2 subjects who showed doubtful reactions to PPD and and did not react to ME‐PPD.

**Table 1:** Overview of the subjects included in the present study on cross‐elicitation reactions to 2‐methoxymethyl‐*p*‐phenylenediamine (ME‐PPD) in *p*‐phenylenediamine (PPD)‐positive individuals

	Total *N* = 25
Original PPD patch test reaction (%)	
+	5 (20.0)
++	12 (48.0)
+++	8 (32.0)
Tested with all 5 ME‐PPD patch test concentrations	23
Tested only with ME‐PPD 0.5%, 1.0%, and 2.0%	2
Gender: male/female	2/23
Age (y), mean (range)	50.2 (18–71)

**Table 2:** Reactions of all 25 *p*‐phenylenediamine (PPD)‐allergic individuals to a 45‐minute open use test with hair dyes containing 2.0% of either PPD or 2‐methoxymethyl‐*p*‐phenylenediamine (ME‐PPD), and patch test results with increasing concentrations of ME‐PPD in petrolatum

		Open use test Hair dye		ME‐PPD Patch test				
		PPD	ME‐PPD					
No.	PPD patch test	2.0%	2.0%	0.1%	0.25%	0.5%	1.0%	2.0%
1	+	+++	++	++	+++	+++	+++	+++
2	+	?+	?+	−	−	+	++	++
3	+	+	−	?+	?+	−	−	−
4	+	+++	−	−	−	−	+	++
5	+	?+	−	−	−	?+	?+	?+
6	++	++	+	+	+	+	+	+
7	++	++	+	++	++	++	+++	++
8	++	+++	+	NT	NT	++	++	+++
9	++	++	−	++	+++	++	+++	+++
10	++	++	+	++	++	++	++	+++
11	++	++	+	?	+	+	++	+++
12	++	++	−	−	−	−	−	+
13	++	?+	−	−	−	−	−	−
14	++	+	−	?+	?+	?+	+	++
15	++	++	+	+	++	++	++	++
16	++	++	?+	−	−	+	+	+
17	++	−	−	−	−	−	−	−
18	+++	+++	+++	NT	NT	+++	+++	+++
19	+++	++	−	−	?+	+	+	+
20	+++	+++	++	+++	+++	+++	+++	+++
21	+++	++	−	+	++	++	++	++
22	+++	+++	−	+	++	++	+++	+++
23	+++	+++	++	++	+++	+++	+++	+++
24	+++	+++	++	+++	+++	+++	+++	+++
25	+++	+++	++	++	++	+++	+++	+++

–, negative patch test reaction; ?+, doubtful patch test reaction, considered to be negative in statistical analyses; NT, not tested.

**Figure 1:**
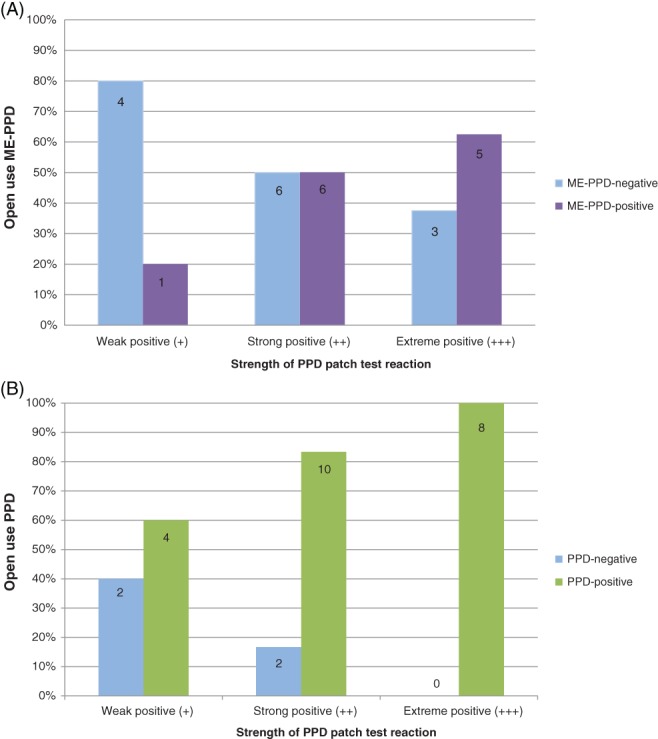
**(A)**, Percentages of *p*‐phenylenediamine (PPD)‐sensitized subjects with either negative or positive reactions to 2.0% 2‐methoxymethyl‐*p*‐phenylenediamine (ME‐PPD) following 45 minutes of exposure in hair dye open use testing, plotted against the strengths of their patch test reactions to PPD. **(B)**, Percentages of PPD‐sensitized subjects with either negative or positive reactions to 2.0% PPD following 45 minutes of exposure in hair dye open use testing, plotted against the strengths of their patch test reactions to PPD

Figure 1A shows the distribution of the non‐responsive subjects and cross‐reacting subjects in ME‐PPD open use testing against the strengths of the original diagnostic patch test reactions to PPD. The percentage of subjects cross‐reacting to the ME‐PPD open use test increased with the strength of the original PPD patch test reaction: 20% (1/5) with +, 50% (6/12) with ++, and 62.5% (5/8) with +++. In contrast, elicitation responses to identical open use tests with 2.0% PPD hair dye were as follows: 60% (3/5) with +, 83.3% (10/12) with ++, and 100% (8/8) with +++ (Figure 1B), giving an elicitation response rate of 84% (21/25) PPD‐allergic subjects.

Furthermore, the reaction strength of the cross‐elicitation response was reduced in 11 of the 12 PPD‐allergic subjects who reacted to both PPD and ME‐PPD open use tests; when plotted against their PPD patch test reaction strengths, reduced cross‐elicitation reaction strengths occurred in all subgroups: 1 of 1 with +, 6 of 6 with ++, and 4 of 5 with +++ (Table 2).

None of the subjects showed immediate reactivity to either ME‐PPD or PPD hair dye open use tests, as no positive reactions were seen 60 minutes after application. No positive reactions to the open use test with the vehicle (negative control) were seen. Eight of the 25 subjects had previously undergone an open use test with ME‐PPD.^7^ None of these 8 subjects had been exposed to ME‐PPD hair dye in any other manner prior to participation in the present study. The 2 who did not react to the ME‐PPD open use test in the previous study did not react to ME‐PPD in the present study, suggesting that they were not actively sensitized in the previous study.

### 3.2 Dose‐dependent cross‐elicitation reactions to ME‐PPD in diagnostic patch testing

**Figure 2:**
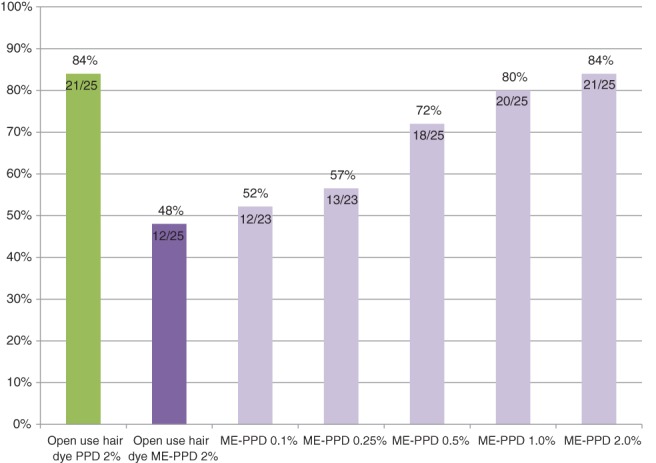
Proportions of response of *p*‐phenylenediamine (PPD)‐sensitized subjects (*n* = 25) to 45 minutes of open use test exposure to hair dye containing 2% of either PPD (green bar) or 2‐methoxymethyl‐*p*‐phenylenediamine (ME‐PPD) (dark violet bar), and to 48 hours of patch test exposure to increasing ME‐PPD concentrations (violet bars)

Figure 2 provides a summary of the relative frequencies of cross‐elicitation reactions to increasing ME‐PPD doses in diagnostic patch testing as compared with the rate of open use cross‐elicitation reactions to 2.0% ME‐PPD and the rate of open use elicitation reactions to 2.0% PPD. Cross‐elicitation responsiveness to increasing concentrations of ME‐PPD (from 0.1% to 2.0% pet.) was tested in PPD‐allergic subjects by patch testing (23 were tested with all concentrations, and 2 were only available for the 3 highest concentrations). The percentage of subjects who showed a positive cross‐elicitation reaction to ME‐PPD 0.1% pet. was 52% (12/23, 95%CI: 32%‐72%), and increased to 84% (21/25, 95%CI: 70%‐98%) when they were exposed to ME‐PPD 2.0% pet. A percentage of 84% also reacted to the open use tests with 2.0% PPD, although these were not exactly the same subjects; of the 21 subjects with an elicitation response to 2.0% ME‐PPD, 20 showed a reaction to the open use test with 2.0% PPD, and vice versa (Table 2). No irritant patch test reactions were seen.

**Figure 3:**
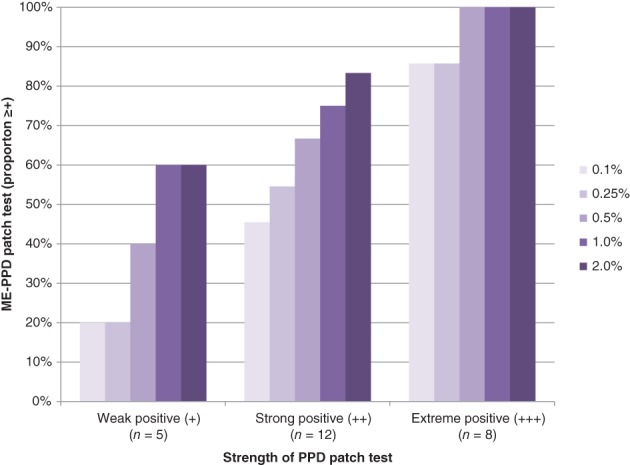
Cross‐elicitation reactions of *p*‐phenylenediamine (PPD)‐allergic subjects to patch testing with increasing 2‐methoxymethyl‐*p*‐phenylenediamine (ME‐PPD) concentrations plotted against the strengths of their patch test reactions to PPD

Figure 3 shows the distribution of the PPD‐allergic subjects with positive cross‐elicitation reactions to the increasing concentrations of ME‐PPD against the strength of the original PPD diagnostic patch test reactions. The data indicate a clear correlation of increasing cross‐reactivity to ME‐PPD and increasing strength of PPD reactivity; that is, all subjects with an extreme (+++) positive reaction to PPD had a positive cross‐elicitation reaction to ME‐PPD at a concentration of 0.5% and above.

**Figure 4:**
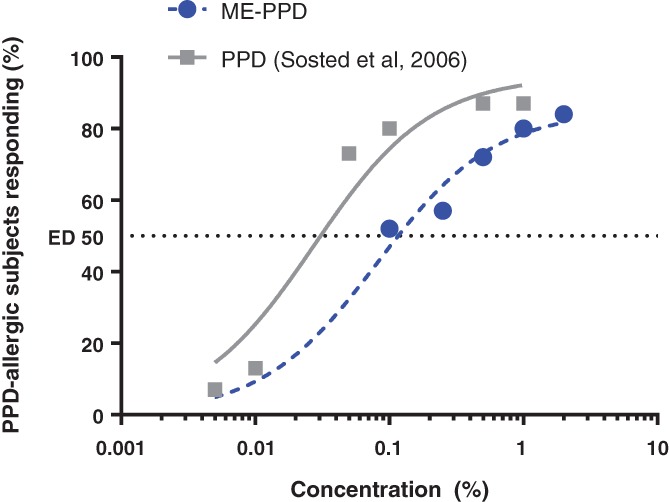
Cross‐elicitation dose‐response curve for 2‐methoxymethyl‐*p*‐phenylenediamine (ME‐PPD) patch testing. The blue line and circles represent the dose response to ME‐PPD in 25 *p*‐phenylenediamine (PPD)‐allergic patients; the grey line and squares represent the dose response to PPD in the 15 PPD‐allergic patients (taken from Søsted et al^5^). Each symbol corresponds to the number of subjects with a positive reaction at the concentration indicated

On the basis of the patch test results with ME‐PPD, a cross‐elicitation dose‐response curve was plotted for the 25 PPD‐allergic subjects (Figure 4, blue dashed line) and compared with a previously published elicitation dose‐response curve for PPD in 15 PPD‐allergic patients (Figure 4, grey line) from Søsted et al.^5^ The curve for ME‐PPD was shifted further towards higher concentrations, indicating that, over the entire concentration range, higher doses are needed to generate a response rate comparable to that of PPD. The expected patch test doses (ED*y* values) at which 50%, 75% and 90% of PPD‐allergic subjects are estimated to develop a positive cross‐reaction to ME‐PPD are shown in Table 3. The ED50 for ME‐PPD was calculated to be ∼0.11%. When we used the data from Søsted et al in our calculation, the ED50 was 0.03% for PPD, in line with the ED50 value reported by Søsted et al.^5^

**Table 3:** Calculated values of the effective dose (ED)*y* for 2‐methoxymethyl‐*p*‐phenylenediamine (ME‐PPD) cross‐elicitation reactions observed in *p*‐phenylenediamine (PPD)‐allergic subjects (*n* = 25) with 95% confidence intervals (CIs) as compared with PPD elicitation dose responses

	ME‐PPD		PPD (Søsted et al)^a^	
	Dose (%)	95%CI (%)	Dose (%)	95%CI (%)
ED50	0.11	0.03‐1.49	0.03	0.01‐0.10
ED75	0.72	0.32‐1.63	0.10	0.04‐0.51
ED90	4.94	0.75‐32.4	0.28	0.10‐3.40

a ED*y* values for PPD 2.0% pet. Patch test data were taken from Søsted et al.^5^ Values were transformed from ppm to %.

## 4 DISCUSSION

In this study, we have analysed cross‐elicitation responses to ME‐PPD in 25 PPD‐allergic individuals by comparing their responses to open use tests simulating hair dyeing, and to diagnostic patch tests with increasing ME‐PPD concentrations.

Open use testing with 2.0% ME‐PPD in a hair dye showed cross‐elicitation in 12 of the 25 PPD‐allergic subjects, that is, in 48% (Figure 1A). In contrast, open use testing with 2.0% PPD (Figure 1B) showed an elicitation response in 84% (21/25). Furthermore, the reaction strength of the cross‐elicitation response was reduced in 11 of the 12 (92%) PPD‐allergic subjects who reacted to both PPD and ME‐PPD open use tests (Table 2). This is in line with data from previous cross‐elicitation studies,^7^, ^10^ indicating partial cross‐reactivity to ME‐PPD under hair dye use conditions. In those previous studies, the response was ∼30% with an exposure time of 30 minutes. The observed higher response of 48% to the 45‐minute ME‐PPD hair dye open use test in the present study confirms previous findings with PPD showing that an increase in the exposure time from 30 to 60 minutes increased the amount available for (cross‐)elicitation by >2‐fold.^11^

We also investigated whether non‐responsiveness to ME‐PPD open use tests in PPD‐allergic subjects is dependent on the limited 45‐minute exposure time. Exposure to increasing ME‐PPD patch test doses for 48 hours under occlusion showed a dose‐dependent increase in the cross‐elicitation response: at the lowest patch test dose of 0.1%, the response of 52% was similar to that with hair dye open use testing with 2.0% ME‐PPD (48%), indicating that a comparable immune stimulus can be provided with either short high‐dose exposure or longer low‐dose exposure. This interpretation is further supported by similar cross‐elicitation reaction strengths in 5 of the 9 subjects (55.0%) who showed elicitation reactions to both 0.1% patch tests and 2.0% open use tests.^12^

With increasing patch test doses up to 2.0%, an increasing number of PPD‐allergic subjects cross‐reacted to ME‐PPD, with 84% showing positive reactions at the maximum tested dose of 2.0% (Figure 1). These findings indicate that the majority of PPD‐allergic patients are probably able to cross‐react to ME‐PPD, provided that the dose is high enough and/or the exposure time is long enough. In line with the responses to open use testing (Figure 1A), a higher percentage of the extreme PPD responders than of the weaker PPD responders cross‐reacted at lower ME‐PPD patch test doses. A possible explanation might be a higher number of PPD‐specific memory T cells being present in the extreme PPD responders.

On comparison of the ME‐PPD cross‐elicitation patch test dose response obtained in the present study with the patch test dose response to PPD obtained in 15 PPD‐allergic subjects,^5^ the curve for ME‐PPD is shifted further towards higher concentrations (Figure 3). In other words, over the entire dose range, higher ME‐PPD concentrations are needed to generate a response rate comparable to that seen with PPD. This is also indicated by the 4‐fold increased ED50 value for ME‐PPD vs PPD, and is in line with the hypothesis that ME‐PPD is a less potent allergen.^3^, ^5^

The measured exposure level (MEL) (representing the concentration that is available to the skin) of the applied PPD patch dose of 1.0% for 48 hours is 200 μg/cm^2^. This is an order of magnitude higher than the MEL for PPD in hair dye open use conditions for 30 minutes, which ranges from 6.8 to 21 μg/cm^2^.^11^ This PPD concentration was found to be sufficient to elicit a reaction in 84% of PPD‐allergic patients. For ME‐PPD, the MELs for 1.8% applied under hair dye use conditions were 8.75 and 10.21 μg/cm^2^ for 30 minutes and 60 minutes, respectively,^3^ and were thus similar to the MEL for PPD. Consequently, it is reasonable to assume that the MEL for patch test conditions with exposure to 2% ME‐PPD is at least >10 fold higher and in line with the higher response observed in patch testing than in open use testing.

In agreement with the present results, a study from Kock et al investigating cross‐elicitation reactions to repeated hair dyeing with ME‐PPD in PPD‐allergic and/or TDA‐allergic individuals showed that 29 of 43 were able to tolerate repeated hair colouring with an average of 9 treatments per year.^13^ In that study, 9 subjects did not react to the initial 45‐minute open use test on the forearm, and 7 of them developed allergic contact dermatitis symptoms during the first hair colouration with ME‐PPD. Together with our findings, these data suggest that an individual threshold for cross‐elicitation to ME‐PPD exists in PPD‐allergic subjects. We assume that the skin site‐specific occurrence of the cross‐elicitation responses to ME‐PPD between forearm and back (in the present study) or scalp (in the study from Kock et al^13^) is related to the presence of (sufficient) residual memory T cells. Similar findings have been reported for patients with allergy to nickel: a rapid response to nickel only occurred after re‐exposure to nickel on the exact body site that had been previously exposed, and the magnitude of the elicitation responses correlated with local recruitment of epidermal CD8^+^ T cells.^14^

In line with previous studies on ME‐PPD, our hair dye open use and patch test data further support the recommendation for hair dye‐allergic individuals to avoid hair colouring, because cross‐elicitation responses to ME‐PPD cannot be excluded.^7^, ^10^, ^13^

Despite intensive investigations, the precise hapten responsible for PPD allergy has not been identified.^15^ Considering that induction of sensitization with PPD has been shown to be dependent on duration of exposure,^16^ PPD oxidation to protein reactive auto‐oxidation products such as Bandrowski's base may be involved in elicitation.^17^ Furthermore, PPD undergoes N‐acetylation when entering the epidermis, and thus becomes deactivated.^18^ In line with the findings for PPD, both oxidative activation of ME‐PPD and deactivation by N‐acetylation have been described in vitro, and human skin has been shown to actively N‐acetylate ME‐PPD.^3^ Correspondingly, the structural similarity to PPD and the consistent activation and deactivation mechanisms suggest that, in PPD‐allergic individuals, the concentration‐dependent immune response to ME‐PPD is based on the partial inability of T cells to differentiate between PPD‐derived and ME‐PPD‐derived haptens.^10^

## 5 CONCLUSION

Cross‐elicitation analysis in PPD‐allergic individuals indicates that ME‐PPD is a less potent allergen than PPD under simulated hair dye use conditions and under diagnostic patch test conditions. The cross‐reactivity shows a clear dose dependency, with increasing cross‐reactivity to ME‐PPD being seen at higher patch test doses. ME‐PPD can only be considered to be an alternative hair dye for primary prevention of sensitization.

### Conflict of interest

We declare that Procter & Gamble Professional Beauty (now represented by Coty) provided funding support for conducting the study and provided the hair dyes. The hair dye ingredient investigated in this study is currently used in commercial products marketed by Coty. C. Goebel is an employee of Coty. The authors alone are responsible for the content and writing of the article.

## References

[1] SCCS (Scientific Committee on Consumer Safety) . Memorandum on hair dye chemical sensitisation. February 26, 2013 https://ec.europa.eu/health/scientific_committees/consumer_safety/docs/sccs_s_007.pdf

[2] SCCS (Scientific Committee on Consumer Safety) . Opinion on: hydroxyethyl‐p‐phenylenediamine sulfate COLIPA no. A80. 2010 https://ec.europa.eu/health/scientific_committees/consumer_safety/docs/sccs_o_017.pdf

[3] Goebel C , Troutman J , Hennen J , et al. Introduction of a methoxymethyl side chain into p‐phenylenediamine attenuates its sensitizing potency and reduces the risk of allergy induction. Toxicol Appl Pharmacol. 2014;274:480‐487. 24333256 10.1016/j.taap.2013.11.016

[4] Ho S , Basketter D , Jefferies D , Rycroft R , White I , McFadden J . Analysis of para‐phenylenediamine allergic patients in relation to strength of patch test reaction. Br J Dermatol. 2005;153:364‐367. 16086750 10.1111/j.1365-2133.2005.06742.x

[5] Søsted H , Menné T , Johansen JD . Patch test dose–response study of p‐phenylenediamine: thresholds and anatomical regional differences. Contact Dermatitis. 2006;54:145‐149. 16524437 10.1111/j.0105-1873.2006.00803.x

[6] Johansen JD , Aalto‐Korte K , Agner T , et al. European Society of Contact Dermatitis guideline for diagnostic patch testing—recommendations on best practice. Contact Dermatitis. 2015;73:195‐221. 26179009 10.1111/cod.12432

[7] Blömeke B , Pot L , Coenraads P , Hennen J , Kock M , Goebel C . Cross‐elicitation responses to 2‐methoxymethyl‐p‐phenylenediamine under hair dye use conditions in p‐phenylenediamine‐allergic individuals. Br J Dermatol. 2015;172:976‐980. 25234507 10.1111/bjd.13412

[8] Scientific Committee on Consumer Products . Opinion on: 2‐Methyl‐5‐Hydroxyethylaminophenol COLIPA no. A31. 2006 https://ec.europa.eu/health/ph_risk/committees/04_sccp/docs/sccp_o_040.pdf

[9] Scientific Committee on Consumer Products . Opinion on: 2‐Methylresorcinol COLIPA no. A44. 2008 https://ec.europa.eu/health/ph_risk/committees/04_sccp/docs/sccp_o_151.pdf

[10] Zahir A , Kindred C , Blomeke B , Goebel C , Gaspari AA . Tolerance to a hair dye product containing 2‐methoxymethyl‐p‐phenylenediamine in an ethnically diverse population of p‐phenylenediamine‐allergic individuals. Dermatitis. 2016;27:355‐361. 27775972 10.1097/DER.0000000000000230

[11] Goebel C , Coenraads P , Rothe H , et al. Elicitation of the immune response to p‐phenylenediamine in allergic patients: the role of dose and exposure time. Br J Dermatol. 2010;163:1205‐1211. 20795999 10.1111/j.1365-2133.2010.10009.x

[12] Friedmann P . The relationships between exposure dose and response in induction and elicitation of contact hypersensitivity in humans. Br J Dermatol. 2007;157:1093‐1102. 17854376 10.1111/j.1365-2133.2007.08162.x

[13] Kock M , Coenraads P , Blömeke B , Goebel C . Continuous usage of a hair dye product containing 2‐methoxymethyl‐para‐phenylenediamine by hair‐dye‐allergic individuals. Br J Dermatol. 2016;174:1042‐1050. 26749506 10.1111/bjd.14390

[14] Schmidt JD , Ahlström MG , Johansen JD , et al. Rapid allergen‐induced interleukin‐17 and interferon‐γ secretion by skin‐resident memory CD8 T cells. Contact Dermatitis. 2017;76:218‐227. 27873334 10.1111/cod.12715

[15] Farrell J , Jenkinson C , Lavergne SN , Maggs JL , Park BK , Naisbitt DJ . Investigation of the immunogenicity of p‐phenylenediamine and Bandrowski's base in the mouse. Toxicol Lett. 2009;185:153‐159. 19136049 10.1016/j.toxlet.2008.12.008

[16] Basketter DA , Jefferies D , Safford BJ , et al. The impact of exposure variables on the induction of skin sensitization. Contact Dermatitis. 2006;55:178‐185. 16918618 10.1111/j.1600-0536.2006.00906.x

[17] Ito A , Nishioka K , Kanto H , et al. A multi‐institutional joint study of contact dermatitis related to hair colouring and perming agents in Japan. Contact Dermatitis. 2017;77:42‐48. 28425114 10.1111/cod.12783

[18] Schuttelaar MA , Vogel TA . Contact allergy to hair dyes. Cosmetics. 2016;3:21.

